# Measurement-Based Domain Parameter Optimization in Electrical Impedance Tomography Imaging

**DOI:** 10.3390/s21072507

**Published:** 2021-04-03

**Authors:** Jan Dusek, Jan Mikulka

**Affiliations:** Department of Theoretical and Experimental Electrical Engineering, Brno University of Technology, 61600 Brno, Czech Republic; mikulka@vut.cz

**Keywords:** electrical impedance tomography, Nelder–Mead optimization, electrode locations, domain deformation, complete electrode model

## Abstract

This paper discusses the optimization of domain parameters in electrical impedance tomography-based imaging. Precise image reconstruction requires accurate, well-correlated physical and numerical finite element method (FEM) models; thus, we employed the Nelder–Mead algorithm and a complete electrode model to evaluate the individual parameters, including the initial conductivity, electrode misplacement, and shape deformation. The optimization process was designed to calculate the parameters of the numerical model before the image reconstruction. The models were verified via simulation and experimental measurement with single source current patterns. The impact of the optimization on the above parameters was reflected in the applied image reconstruction process, where the conductivity error dropped by 6.16% and 11.58% in adjacent and opposite driving, respectively. In the shape deformation, the inhomogeneity area ratio increased by 11.0% and 48.9%; the imprecise placement of the 6th electrode was successfully optimized with adjacent driving; the conductivity error dropped by 12.69%; and the inhomogeneity localization exhibited a rise of 66.7%. The opposite driving option produces undesired duality resulting from the measurement pattern. The designed optimization process proved to be suitable for correlating the numerical and the physical models, and it also enabled us to eliminate imaging uncertainties and artifacts.

## 1. Introduction

Electrical impedance tomography (EIT) is a non-invasive method to deliver cross-sectional images. The non-invasive character, available cost, portability, and safe use have allowed the technique to find wide application in diverse branches and disciplines of industry and science, such as biomedicine [1,2,3], geophysics [4,5,6,7], industrial chemistry [8,9,10,11], and material engineering [12,13,14,15].

Principally, the approach relies on exciting the examined object by a harmonic current that passes through electrodes on its perimeter and, simultaneously, measuring the electric potential on the remaining electrodes according to the selected current pattern. The obtained values of the voltage or the phase difference between the current and the voltage (allowing admittivity reconstruction) enable us to reconstruct the specific conductivity distribution. In general terms, the presented reconstruction process can be described as a nonlinear and ill-posed inverse task. Considering the sensitivity of the process to changes in the individual parameters (the geometrical accuracy of the domain and positioning of the electrodes), the numerical finite element method (FEM)-based model of the domain must be set up in a precise manner. The various inaccuracies are advantageously reduced via optimizing the parameters of the forward solution model; such a step is beneficial because the inverse task, due to its character, does not enable us to estimate the parameters accurately. The solution is exactly defined by a physical and a numerical model, and its results should correlate with those of the real measurement. Precise domain modeling was previously proven to be a necessary precondition for high-quality image reconstruction. References [16,17], for example, showed that an inaccurate shape of the domain markedly affects the measured voltages, the impact being even greater than that exerted by the actual inhomogeneities. The deformed shape results in the blurring and incorrect localization or inferior recognizability of an inhomogeneity [18]. Regarding the current patterns, the problem occurs more frequently in single-source than in trigonometric driving [17]. The domain parameters were investigated in detail by, for instance, Hemant Jain et al. [19], who examined the impact of non-circular boundaries on the reconstruction of comprehensive specific conductivity via the NOSER algorithm. During their experiments, the elliptically deformed circular domain caused the reconstructed image to exhibit substantial distortion, meaning that precise domain modeling has a fundamental influence on the reconstruction accuracy. Furthermore, the conductivity value error proved to be significantly lower in the axis than on the boundary of the investigated object.

Imprecise modeling and the distribution of the electrodes were discussed in reference [17], with the outcome being that electrode misplacement may introduce in the reconstruction an error larger than the uncertainty of the entire measuring system. This type of inaccuracy results in blurring, artifacts, and incorrect localization; such spurious effects are more frequently encountered in single-source driving than in the trigonometric pattern and can be suppressed through accentuating regularization at the expense of losing the absolute value of conductivity. The same or associated problems, i.e., modeling inaccuracies, the positioning of the electrodes, and the measured voltage and current errors, were analyzed by Murphy et al. in [20]. The authors examined the impact of the individual parameters on EIT reconstruction via the D-Bar approach, which allows one to solve the inverse task without iterative computation. The evaluation was carried out through simulation, exclusive of measurement, and concluded that incorrect modeling eventually generates artifacts.

The influences exerted by inappropriate electrode surface and contact impedance on the reconstruction process were focused on in references [17,18]. In addition to showing that electrode inaccuracies affect the reconstructed image to a markedly greater extent in multi-source than in single-source driving, the research also confirmed that the electrode size modeling error is indirectly proportional to the contact impedance deviation; thus, overestimating the electrode surfaces brings an effect similar to that of underestimating the contact impedance. From the perspective of measurement, the inaccuracy of the reconstructed image, given by the contact impedance, resembles in character an incorrectly set surface of the electrodes: while the issue will not manifest itself in neighboring patterns, in trigonometric driving, which comprises current-carrying electrodes, it substantially contributes to the formation of ring artifacts to impair the accuracy of the image.

In view of the higher signal-to-noise ratio (SNR) in the trigonometric pattern, a dedicated article presented the possibilities of compensating for contact impedance via triple measurement or a suitable structure with compound electrodes [21]. By extension, other papers [22,23] centered on the effect of inaccurately known contact impedances, addressing the issue of contact impedance estimation based on the complete electrode model (CEM). For reliability purposes, the estimation was compared with measurement performed by means of an Oxford Brookes and a KIT4 tomograph; the distribution of the conductivity and contact impedance was reconstructed with real data. Finally, the results of the experiments showed that the contact impedance cannot be separated from the internal impedivity and estimated without measurement utilizing a uniform conducting medium.

In relation to clinical use, the problem of an unknown domain border and contact impedance was investigated in [18]. The proposed novel approach challenged the inaccuracies numerically to successfully reduce the systematic errors in the reconstruction, with the original reconstructing procedure based on measurement; the deformation case, however, was verified solely via simulation.

The compensation of variable electrode contact in EIT was introduced in [24], where the authors employed the CEM to demonstrate a hybrid nonlinear reconstruction algorithm that allows the substantial reduction of artifacts generated through poor contact. The compensation method was tested on a set of clinical data. Another compensation technique for modeling the unknown domain boundary error was presented in [25]. This instrument exploits the approximation approach, and its efficiency was validated by experimental measurement on an EIT phantom. In [26], fast and simultaneous statistical estimation of conductivity and electrode contact impedances for a 2D disc was outlined; the method fundamentally utilized the Toeplitz matrices to identify wrong contacts. The described approach proved operable and was verified via measurement.

The optimization of the electrode position in irregularly shaped domains was examined in [27,28], relying on an optimizing algorithm of the steepest descent type. Exploiting Fréchet derivatives of the CEM, the authors computed the electrode locations through simulation. In [29], a procedure to compensate for imprecise electrode modeling and movement artifacts was described in detail, together with surface movement reconstruction options. The compensation was tested with the EIDORS tool, and the reconstruction exploited a real or a reproduced data set. The research characterized in [30] targeted simultaneous reconstruction of time-varying images and contact impedance, yielding a significantly reduced electrode drift and motion artifacts. The method was evaluated on reconstructed clinical data, measured by using a GE GENESIS prototype.

A 2D-based procedure for optimizing the electrode position by means of deep learning was outlined in paper [31]. The experiment involved simulating voltage data with added Gaussian noise upon 1%, 5%, and 10% standard deviation, and performing image reconstructions on non-circular domain shapes. The researchers concluded that optimized electrode positions can reduce the error in EIT reconstructions; as regards the experimental input, however, they employed only synthetic data, meaning that factors accompanying real measurement, such as interference, uncertainties of the instruments, and limited resolution, were not assumed. Further, the size and polynomial degree of the mesh elements gained attention in report [32], which examined the relationship between the mesh density and the resulting error. The authors proposed that a higher density does not necessarily yield a lower reconstruction error, and they also specified the key variables causing unexpected behavior in the inverse task.

As pointed out above, our project comprised not only numerical modeling and simulation but also laboratory measurements. Utilizing the obtained data and knowledge, we set up an optimization procedure based on the Nelder–Mead algorithm; the input is the vector of measured voltages in a real-world tomograph, and the output consists of the approximate value of one or more required parameters (shape deformation of the domain, electrode placement, and initial conductivity). Principally, the actual procedure involves verifying the physical model of the tomograph via computing an FEM-based numerical model, in such a manner that we obtain the best possible match between the resulting vectors of simulated and measured voltages. The optimization was implemented with the EIDORS library and the relevant Matlab toolbox, the aim being to correlate the numerical model and the real-world measurements. The entire set of tasks has been designed to improve the image reconstruction accuracy and to reduce the artifacts. In more general terms, this article contributes to the state of the art by introducing a novel approach to optimize the parameters of the mathematical model, such as the initial conductivity, electrode positions, and domain shape deformations; importantly, the entire procedure is supported by the measurement of the physical model. The research comprises, above all, laboratory experiments and their analysis to improve the applicability of the tomography in the real-world monitoring of large-sized objects. We assume broader and deeper cooperation with the Department of Water Structures, Faculty of Civil Engineering, Brno University of Technology; in this context, the optimized mathematical model will be employed in monitoring the functionality of dams and predicting their material composition.

## 2. Materials and Methods

This section outlines the measurement patterns and the Precise, Low Impedance (PLI) EIT prototype device for data acquisition. In this context, the forward and inverse tasks are derived and described; we also show the Nelder–Mead algorithm with an implementation in computable unknown EIT parameters and demonstrate image reconstruction to develop relevant comprehensive discussion within the last chapter.

### 2.1. Measurement and Device

**Figure 1:**
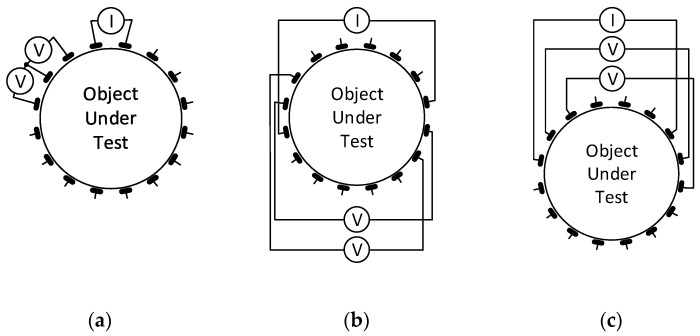
The initial procedural steps in two current pattern sequences for tomographic measurement: (**a**) adjacent; (**b**) opposite; (**c**) skip-5.

The principle of acquiring EIT image reconstruction data exploits voltage measurement and signal phase shift with respect to the input current on the electrodes of the tomograph. Thus, multiple stimulation patterns have been created over the decades, allowing the application of alternating current to predefined electrodes of the system; simultaneously, suitable combinations of electrodes to facilitate the measurement are specified. The most popular single source stimulation patterns include Adjacent, Opposite, and Skip-X driving, all of which are shown in Figure 1 [33,34,35].

However, a multi-source pattern category is also available, with the best known element being the trigonometric current pattern. Each driving option exhibits certain advantages and drawbacks. The benefits of adjacent driving consist of good edge sensitivity and contact impedance insensitivity; conversely, the disadvantages include poor sensitivity at the center; sensitivity to the boundary shape and electrode position; high measurement error; and electrode noise interference. Similarly, the opposite pattern is characterized by good overall performance and uniform current distribution, but also poor edge sensitivity and low current injection. Compared to single-source driving, the trigonometric pattern has strengths such as sufficient center sensitivity, high current injection, and improved SNR; the weaknesses are then embodied in the more independent current drivers and considerable susceptibility to unknown contact impedance [17,36].

**Figure 2:**
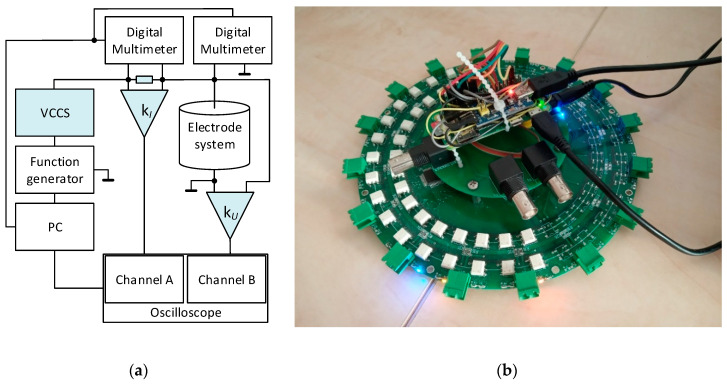
(**a**) The diagram describing the acquisition of the voltage and phase shift data to allow the conductivity reconstruction; (**b**) the prototype of the PLI EIT system (first version).

Utilizing our knowledge of driving patterns, we designed a single-source precise, low impedance EIT system for tomographic data acquisition; the entire setup is presented in greater detail within [37]. The images below display a tomographic data processing diagram and a version 1 EIT switching card, whose components are represented in the drawing (Figure 2).

The designed diagram consists of a function generator and a voltage-controlled current source (VCCS) to deliver a constant AC current amplitude for the stimulation. The feeding part is connected to a shunt resistance Z_B_, which operates as an AC current detector. The shunt resistance is joined to the electrode system of the tomograph through a PLI EIT switch (Figure 2b). The electrode system and shunt resistance are connected with differential amplifiers and digital multimeters to perform the voltage measurement. The amplified voltage drops in the current sensor, and on the tomograph electrodes are evaluated with an oscilloscope to determine the phase shifts. The oscilloscope, multimeters, and function generator are driven by a computer in LabView. The measurement is fully automatic, and the combination of the electrodes is programmable as desired [37].

Figure 2b displays a prototype of the switch, independently extendable and designed with respect to a low on-state impedance (max 0.7 Ω in the range from 10 Hz to 100 kHz). The PLI EIT system also contains the VCCS with a range of ±15 V; two differential amplifiers (AD 620) with a shunt resistance for current sensing; and a battery power supply for the amplifiers. The innovated variant of the PLI EIT system will contain an analog-to-digital converter (ADC) to replace the digital multimeters, and also hysteresis comparators with a counter to substitute for the oscilloscope; currently, this version is being developed [37].

In the actual optimization, we employed only the module of voltages, expecting the voltage values to be equal to the impedivity module. The impact of the signal phase shift will be explored within the future research.

### 2.2. Forward Task

The physical model of the forward task is based on a description of the domain Ω in an R^N^, N = 1, 2, 3 sized space. Let us assume that the domain has smooth, continuous boundaries, to which are connected *L* electrodes equidistantly distributed on the domain’s surface. An alternating current *I_l_* is supplied via the electrodes to the actual domain; when passing through, the current then induces corresponding voltage drops, and these are detected by the electrodes. The forward task, current supply, and measurement of the voltage drops can all be most accurately defined by the complete electrode model (CEM), describable as follows:

(1) −∇(σ(x)∇u(x))=0, x∈Ω,

(2) $$u\left(x\right)+z_{l}\sigma \left(x\right)\frac{\partial u\left(x\right)}{\partial n}=U_{l},\;x\in e_{l},\;l=1,\;\ldots ,\;L,$$

(3) $$\int_{e_{l}}\sigma \left(x\right)\frac{\partial u\left(x\right)}{\partial n}dS=I_{l},\;l=1,\;\ldots ,\;L,$$

(4) $$\sigma \left(x\right)\frac{\partial u\left(x\right)}{\partial n}=0,\;x\in \partial \Omega \backslash \overset{L}{\underset{l=1}{\cup }}e_{l},$$

(5) $$\sum_{l=1}^{L}I_{l}=0,$$

(6) $$\sum_{l=1}^{L}U_{l}=0,$$

where *x* is a coordinate belonging to the domain Ω, *σ*(*x*) denotes the specific conductivity of the investigated medium, *u*(*x*) represents the electric potential inside the domain Ω, *U_l_* and *I_l_* stand for the voltage and current passing through an electrode *l*, *z_l_* is the contact impedance between an electrode and unknown conductivity, and *n* characterizes the vector that is normal to the surface of the domain ∂Ω. The last two of the equations describe Kirchhoff’s laws, or, by extension, the law of the conservation of energy, in a physical expression of the forward task [38,39].

The numerical solution of the above-defined physical model exploits discretization. The partial differential equations (PDEs) are then approximated via the finite element method (FEM). Using the FEM to discretize the domain is regulated by the following formula:

(7) $$u\left(x\right)=\sum_{i}\sum_{j}u_{i,j}N_{i,j}(x),$$

where *u*(*x*) denotes the voltage at any point in the system, *u_i,j_* are the voltages at the nodal points of the mesh, and *N_i,j_* represents the linear basis approximation function relating to the created mesh.

With the finite element method, the equation system of the physical model (CEM) (1)–(4) can be assembled into the form:

(8) Ax=b

(9) $$\left[\begin{array}{cc} G+B & C\\ C^{T} & D \end{array}\right]\left[\begin{array}{c} u_{i}\\ u_{j} \end{array}\right]=\left[\begin{array}{c} 0\\ b \end{array}\right]$$

where the individual elements of the matrix are expressed by the following relationships:

(10) $$G_{i,j}=\int_{\Omega }\sigma \;\nabla N_{i}\cdot \nabla N_{j},$$

(11) $$B_{i,j}=\sum_{l=1}^{L}\int_{E_{l}}N_{i}N_{j},$$

(12) $$C_{i,j}=-\frac{1}{z_{l}}\int_{E_{l}}N_{j},$$

(13) $$D_{i,j}=-\frac{\left|E_{l}\right|}{z_{l}}\;\mathrm{for}\;i\;=\;j\;=\;l;\mathrm{if}\;\mathrm{not},\;\mathrm{then}\;0,\;$$

where *N* denotes the linear approximation function, *σ* represents the conductivity, *z_l_* is the contact impedance, and *E_l_* defines the electrode surface area. The derivation of the system of equations is described in detail within [29,39].

### 2.3. Inverse Task

In general terms, the inverse task is characterized as a difficult-to-solve mathematical function. Within EIT, the operation consists of estimating computationally the distribution of specific conductivity in a domain Ω; the actual computation is nonlinear, ill-posed, and problematic to execute. There are two essential approaches to the EIT inverse solution: difference and absolute imaging. The former procedure reconstructs two diverse measurements, either in time (time-difference) or depending on the frequency (frequency-difference); these measurements are then utilized for computing the relative change of the distribution of conductivity inside the domain. By comparison, the latter option, or absolute imaging, relies on reconstructing the conductivity via a single measurement [3].

Principally, the inverse task rests in searching for such a conductivity matrix that will satisfy the conditions of Equation (1) while preserving the values of the voltage and current within the domain Ω. In EIT, we have the equation:

(14) $$\Psi (\sigma )=A^{†}b,$$

where Ψ(σ) is the conductivity change vector, **A** denotes the Jacobian, and **b** represents the voltage error [39].

By applying the Moore–Penrose inverse, we yield the equation:

(15) $$\Psi (\sigma )=A^{†}b={\left(A^{T}A\right)}^{-1}A^{T}b,$$

which resolves with the least squares method formula:

(16) $$\Psi (\sigma )=\min_{x}\left\|Ax-b\right\|.$$

In the ill-posed matrix **A**, the least squares procedure may fail to perform properly while the inverse task is being solved, rendering the problem unresolvable. Thus, the method was complemented with regularization, exploiting options such as the Tikhonov approach, where the L2 term is applied to penalize (suppress) sharp changes to a greater extent than possible with the L1 term. The least squares method including the Tikhonov regularization is characterized as:

(17) $$\Psi (\sigma )=\min_{x}{\left\|Ax-b\right\|}_{}^{2}+\alpha^{2}{\left\|x\right\|}_{}^{2},$$

where α denotes the regularization parameter [29].

By applying the above functional directly to the inverse task, we yield the objective function formula:

(18) $$\Psi (\sigma )=\min_{x}\frac{1}{2}{\left\|U_{M}-U_{FEM}(\sigma )\right\|}_{}^{2}+\alpha^{2}{\left\|R\sigma \right\|}_{}^{2},$$

where **U**_M_ is the vector of the voltages measured at the border of the domain Ω; **U**_FEM_(σ) represents the vector of the voltages on the electrodes, obtained via the forward solution; *α* stands for the regularization parameter; and **R** denotes the regularization matrix.

The algorithm to iteratively compute the conductivity by utilizing the Newton–Raphson method is obtained through the original estimation, which follows from the Taylor development, assuming that the conditions of the Euler–Lagrange equation have been satisfied to allow Equation (18) to be zero. The iterative conductivity calculation can read:

(19) $$\sigma_{i+1}=\sigma_{i}+\varepsilon ,$$

where *σ_i_*_+1_ is the new approximation of the desired conductivity, *σ_i_* represents the approximation in the previous iteration, and *ε* can be written as:

(20) $$\varepsilon ={\left(J_{i}^{T}J_{i}^{}+\alpha R^{T}R\right)}^{-1}\cdot \left[J_{i}^{T}(U_{M}-U_{\mathrm{FEM}}(\sigma ))+\alpha R^{T}R\sigma_{i}\right],$$

where **J** is the Jacobian, expressing the sensitivity of the discretized electrical potential over the FEM domain model [39,40,41,42,43].

### 2.4. Optimization

The optimization of the parameters (namely, the geometry of the model; regularity, and electrode placement) of the domain fundamentally influences the image reconstruction, as already proposed within the introductory section of the paper. The problem remains very topical because specifying and determining the parameters are critical steps in the effort to achieve more accurate results, reduce the image artifacts, and limit the computational intensity; all of the goals are reached by decreasing the number of the degrees of freedom to be determined through the inverse task. In this context, it is then more advantageous to define as many parameters as possible via pre-calculation before initiating the inverse task.

**Figure 3:**
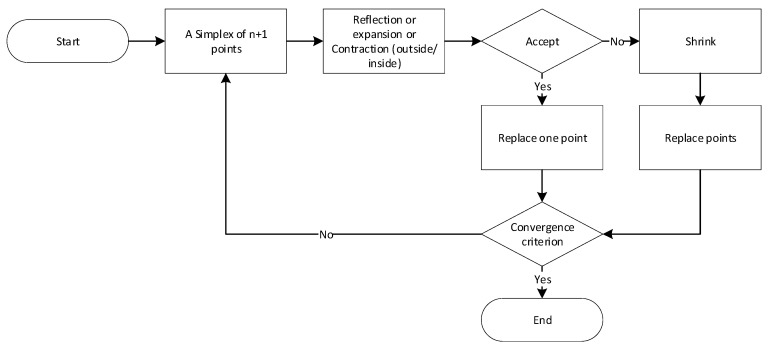
The flowchart of the Nelder–Mead simplex method.

The actual procedure involves verifying the physical model of the tomograph via computing an FEM-based numerical model to calculate the best possible match between the vectors obtained from forward task-simulated and measured voltages. To finally and finely optimize the above-listed parameters, exploiting the measured values, we employed the Nelder–Mead simplex algorithm (Figure 3). Included in the Matlab optimization toolbox, this heuristically based method finds frequent use in solving nonlinear optimization problems where the function derivative can be unknown; in our case, the focus is on the relationship between the measured quantities and the properties of the domain.

When operating, the algorithm utilizes the geometrical structure referred to as the simplex. The structure is a geometrical shape in an R^n^ space with n + 1 points; of these, one point defines the origin of the simplex, while the others determine the direction of the R^n^ space vector. By setting the point of origin, we can generate *n* further points through geometrical transformations (reflection, expansion, and outside/inside contraction). Depending on the transformation applied, the simplex moves, expands, or contracts; moreover, after each transformation, the worst vertex is replaced with a better one. The main advantage of the algorithm over alternative optimization methods rests in its easy implementability, ensuring a highly effective, rapid search for the local optimum [44,45].

The algorithm is initiated by selecting the first point, which then enables the formation of an n‑dimensional topology. Subsequently, the individual vertices, x_1_ to x_n + 1_, are arranged according to the optimization function gradient, f(x_1_) to f(x_n + 1_), with f(x_1_) and f(x_n + 1_) being the best and worst points of the function, respectively. The result of an iteration consists of a new point to substitute for the worst point of the function in the following iteration. If shrinking occurs, the substitution proceeds with *n* new points, which, together with x_1_, are the input simplex points of the following iteration [44,45,46].

To optimize by using the Nelder–Mead algorithm, we defined the minimization function as:

(21) $$\;f(p)=\frac{1}{2}{\left\|U_{M}-U_{\mathrm{FEM}}(p)\right\|}^{2},$$

where *f*(*p*) is the minimization term of the least squares method, *U*_M_ represents the vector of the voltages measured on the laboratory tomograph, and *U*_FEM_(*p*) denotes the voltage on the electrodes evaluated via the forward solution, which is parameterized by the variable *p*.

**Figure 4:**
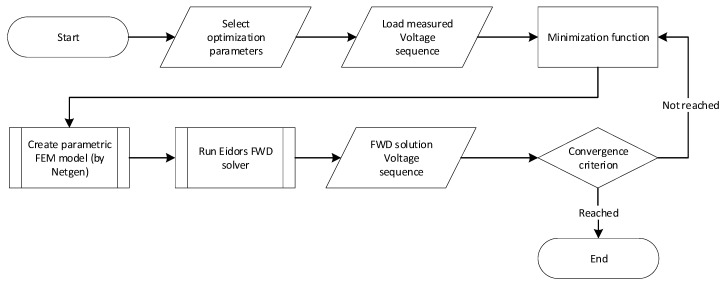
The EIT optimization flowchart based on the measured dataset and selected parameters.

The implementation stages of the algorithm are set out in the diagram below (Figure 4).

The optimization input data consist of one or more selected parameters, including:

- Parametric deformation of the domain (in our case, circular/elliptical deformation);
- Calculation of the initial conductivity;
- Misplacement of the electrodes on the border of the domain.

In addition to the selected optimization parameter, the algorithm also comprises a vector of measured voltages, this being a measured sequence for a particular combination of non-excited electrodes. Based on the preset parameters and the values obtained from the tomograph, the optimization facilitates the generation of a parametric FEM model via Netgen; the model is then solved via the forward task by utilizing EIDORS library [47]. The solution procedure yields a vector of the voltages that were obtained through the simulation, and this vector is evaluated by means of the optimization. If the sum of squares of the differences is greater than the convergence criterion, the algorithm will begin generating a new FEM model and solving the forward task. If the convergence criterion has been satisfied, the optimization will stop, and the return value of the function will contain the nearest possible value of the parameter preset by the user within the selected tolerance.

Considering the large number of parameters, gradual optimization appears to be convenient. Generally, it holds true that the more unknowns at the input of the optimization, the longer the computational time; by extension, we can also point out that if the optimization parameters are set simultaneously, the algorithm may not find a correct solution. To verify the functionality of the algorithm, we used a laboratory model containing a homogeneous medium; this model allowed us to confirm the results physically. This approach then facilitates the determination of factors such as shape deformation, area size, and irregularity in the placement of the electrodes, thus simplifying the subsequent solution of the remaining parameters (initial conductivity and, possibly, contact impedance). The actual impact of the parameters is demonstrated on the results of the reconstruction involving inhomogeneities.

## 3. Experiment

**Figure 5:**
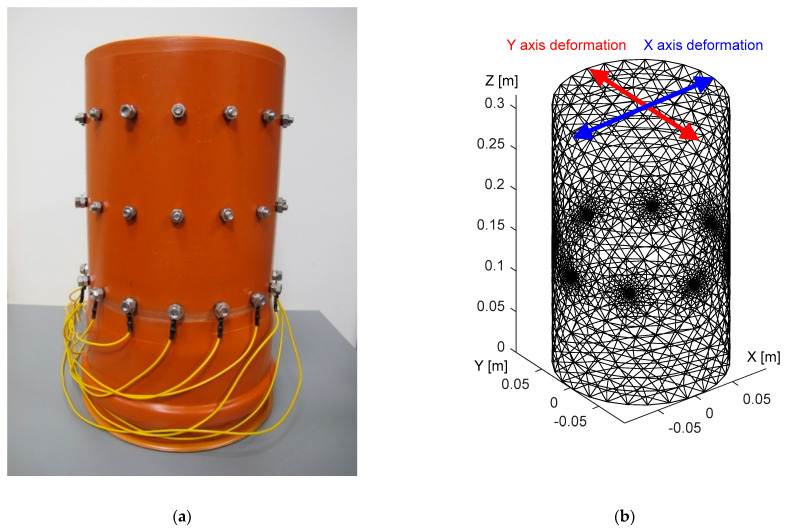
The images: (**a**) the laboratory setup; (**b**) the Netgen-based numerical model.

The experiment was carried out on a laboratory tomograph, supported by a corresponding numerical model of the domain (Figure 5).

The tomograph (Figure 5a), fabricated from polyvinyl chloride, carries electrodes uniformly distributed at three levels along the perimeter. The individual levels are located at 13.6, 21.6, and 29.6 cm above the bottom plane of the vessel. Each stage contains 16 electrodes, namely, stainless steel bolts, and each of the electrodes has a diameter of 9 mm and a height of 6 mm. The diameter and height of the tomograph are 19 and 35.5 cm, respectively.

To evaluate the irregular placement of the electrodes, we set up an 8-electrode configuration (at the top level) and created a corresponding FEM-based model (Figure 5b), which contained approximately 15,000 elements. The remaining, unused electrodes then allowed us to materialize the irregular placement along the perimeter.

### 3.1. Initial Conductivity

**Figure 6:**
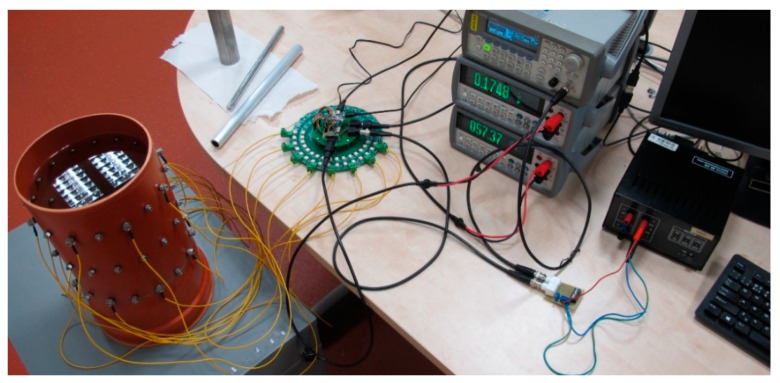
The workstation for the EIT conductivity evaluation.

**Figure 7:**
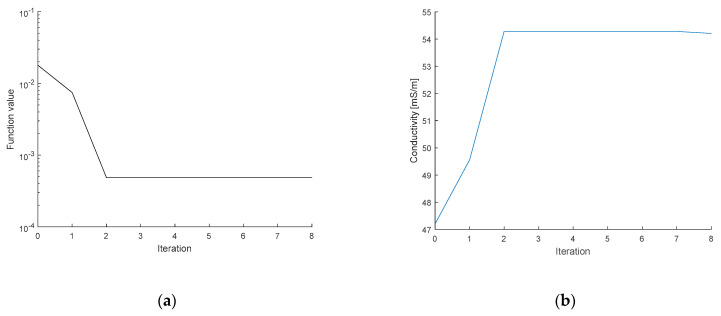
The pattern defining the (**a**) minimization function, and (**b**) the conductivity value during the optimization.

To optimize the initial conductivity, we employed potable water as the medium. The vector of measured voltages was acquired for a combination of two current patterns (adjacent and opposite) and two shapes of the tomograph (a regular circle and a deformation of approximately 2%). The applied FEM model exhibited a height of 0.316 m (water level) and a regular diameter of 0.19 m; the diameters on the axes X and Y of the deformed circular shape corresponded to 0.194 and 0.186 m, respectively; the electrodes were placed at the level of 0.296 m; and the contact impedance equaled 10 mΩ (the EIDORS default value). In the single source patterns, the contact impedance has a negligible effect due to the low current on the measuring electrodes. The input current was 2.002 mA at the frequency of 1007 Hz. The initial conductivity value of 47.2 mS/m was measured with a Total Dissolved Solids (TDS) conductometer. The workstation for the EIT data acquisition is shown in Figure 6; the progress of and changes in the conductivity value and the minimization function are shown in Figure 7a,b, respectively.

In Figure 7, image (a) shows the pattern characterizing the minimization function value during the optimization, while image (b) represents the conductivity at an iteration. The series of the conductivity estimates is very close to the real value at the 3rd iteration. If the novel simplex did not perform better than the previous one, the minimization function will reach the criterion, and the optimization process will stop. The results of the initial conductivity for the experiments characterized in this chapter are shown in Table 1.

Based on the data from Table 1, we obtained the initial conductivity for the experiments. The optimization process took 7 s at the maximum, while the mesh generation time amounted to 3.5 s.

### 3.2. Shape Deformation

**Figure 8:**
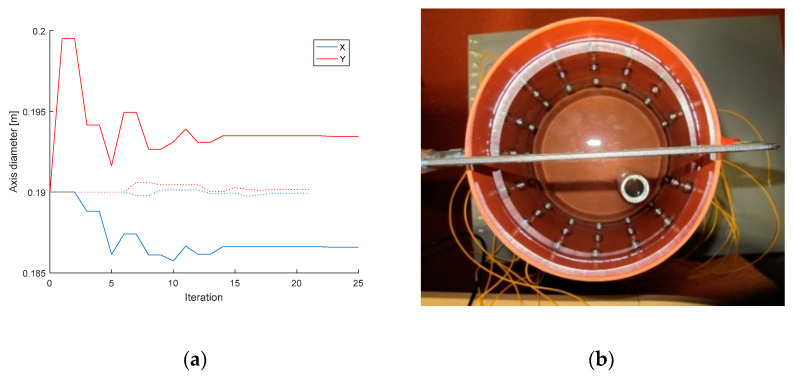
(**a**) The variation of the diameters on the axes X, Y, relating to the opposite stimulation pattern; solid lines and dotted lines show diameter convergence of accurate circular domain shape and non-optimized initial values of elliptical shape, respectively; (**b**) the tomograph filled with water and containing a circular aluminum object.

Shape deformation affects all types of current patterns, as already mentioned in the introductory chapter. To avoid this problem, we designed an applicable optimization procedure; the experiment was performed with the same setup as that presented in Section 3.1. The shape deformation can be evaluated if the diameters on the axes X and Y have been optimized. The circle has the diameter of 19 cm, which is the initial value. Figure 8 below shows the variation of the diameters X, Y during the optimization of the shape domain and also displays the inhomogeneous reconstruction setup.

The shape deformation (Figure 8a) was calculated from the homogeneous measurement, which correlated with the voltages obtained from the forward task. In the case of adjacent driving, the optimization-based axis diameter estimates equaled X = 18.62 cm and Y = 19.35 cm. The opposite pattern then yielded the estimates of X = 18.66 cm and Y = 19.35 cm, both values being very close to the real axis dimensions [18.6; 19.4]. The inaccuracy of the results may have arisen from the voltage uncertainty or insufficient precision of the diameter measurement. The evaluation of the deformation lasted 205 to 250 s. The optimized values of the diameters constituted one of the inputs to enable reconstruction by utilizing laboratory measurement of water with an inserted aluminum object (Figure 8b).

**Figure 9:**
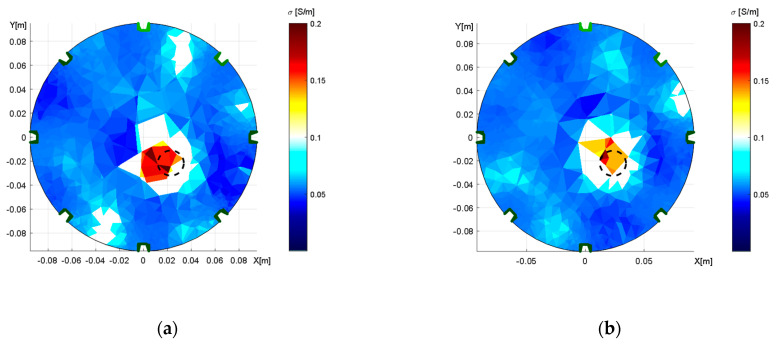
The distribution of conductivity in the water medium, obtained through adjacent driving; an aluminum object is inserted, and the tomograph’s axial dimensions correspond to X = 19.4 cm and Y = 18.6 cm. The images: (**a**) a wrongly selected circular shape; (**b**) the correct elliptic domain.

**Figure 10:**
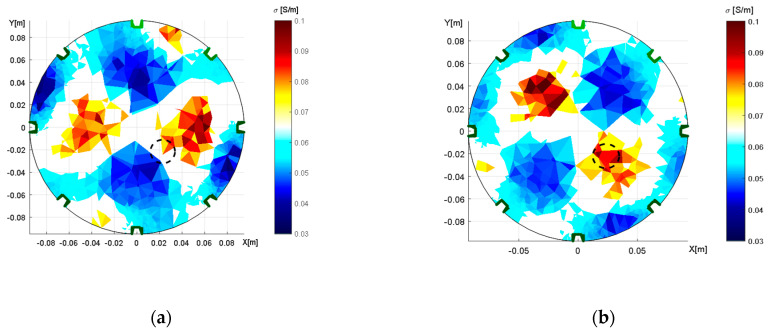
The distribution of conductivity in the water medium, obtained through opposite feeding and sensing; an aluminum object is inserted, and the tomograph’s axial dimensions correspond to X = 19.4 cm and Y = 18.6 cm. The images: (**a**) a wrongly selected circular shape; (**b**) the correct elliptic domain.

Figure 9 and Figure 10 below demonstrate the impact exerted by shape deformation on the reconstructed image in adjacent and opposite driving. The initial conductivity was selected with respect to Table 1; the regularization parameter was set to 0.001.

The reconstructed conductivity distribution shows the importance of accurate domain modeling. It is obvious that the actual modeling caused an incorrect localization (Figure 9a) and that the optimized model (Figure 9b) enabled us to mitigate the drawback in the inhomogeneous object; despite this improvement, however, the result was still not completely perfect. Such an outcome may have been induced by the limitations of the mesh generator, which allowed us to produce deformations only in the *X*- and *Y*-axes. Thus, it could be interesting to prepare the optimization with a more parameterizable deformation of the model.

The conductivity distributions acquired with opposite feeding and sensing were affected by shape deformation to a greater extent than those yielded via the alternative option. The incorrect modeling of the domain (Figure 10a) caused the inhomogeneous areas to rotate, this being an effect that had not occurred in the correct elliptic model (Figure 10b). Each of the reconstructed images contains two regions with a higher conductivity distribution, and these regions correspond to the original and the artefact of the object. Such an arrangement stems from opposite sensing because each scheduled pair of electrodes is measured twice at different polarities, depending on the measuring configuration; the duplicity nevertheless impeded the suppression of the mirror object as was also discussed in [48,49]. The possible solutions to exclude the artifacts are an additional measurement to complement the opposite sensing or selection of different measuring configurations.

### 3.3. Misplacement of the Electrodes

**Figure 11:**
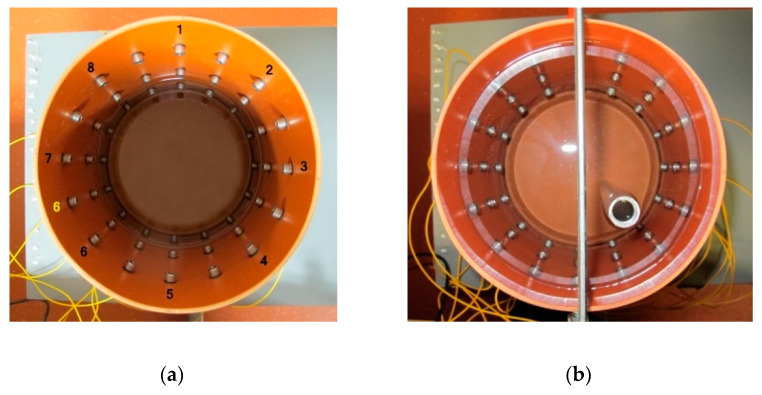
The experimental setup to induce electrode misplacement: (**a**) the shifted 6th electrode; (**b**) the tomograph (19 × 19 cm^2^) filled with water and containing an aluminum object.

**Figure 12:**
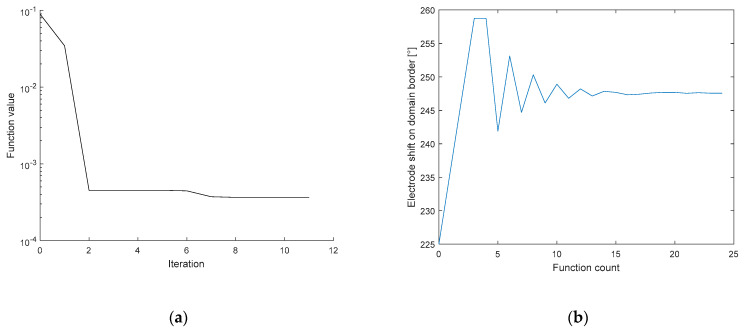
The optimization of the 6th electrode shifts on the domain border, represented with respect to the (**a**) error related to the position of the electrode, varying with iteration; (**b**) function count.

Another source of image reconstruction error is the misplacement of the electrodes. To explore this problem, we conducted an experiment with a misplaced 6th electrode located near the 7th one (Figure 11a). The correct positions of the electrodes were identified by optimizing the homogeneous medium and reconstructing with an inhomogeneous object (Figure 11b). Figure 12 below visualizes the optimization steps for each iteration and also displays the changing position of the electrode related to the function count obtained via simplex.

**Figure 13:**
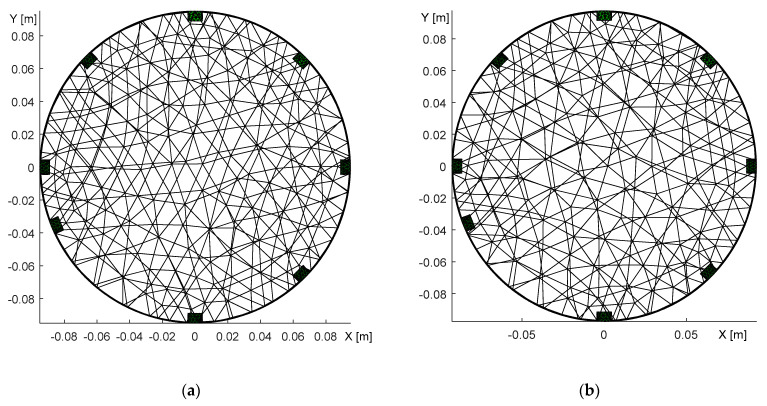
A top view of the optimized model, with the electrode shift obtained through adjacent driving: (**a**) the circular domain; (**b**) the elliptic domain.

Figure 12a depicts the error in relation to the position of the electrode (shift); the image shows that the correct angle is almost reached in the 3rd iteration. By comparison, Figure 12b represents the electrode shift with respect to the changing value of the angle during the function count, i.e., the count of the optimization operations. Each optimization iteration comprises several steps, as explained in Section 2.4. The electrode was shifted along the boundary from 225° to 260°, eventually converging to 247.6°, where the novel simplex did not perform better than the previous one. The process to optimize the electrode misplacement took between 90 and 110 s. The correct positions of the electrodes for the circular and the elliptic domains are shown in Figure 13.

The domain models representing the electrode movement (Figure 13) were generated from the dataset obtained via adjacent driving, utilizing the same experimental setup as that described in Section 3.1. The 6th electrode was relocated to the position indicated in Figure 11a, where the desired location is highlighted with the yellow number.

**Figure 14:**
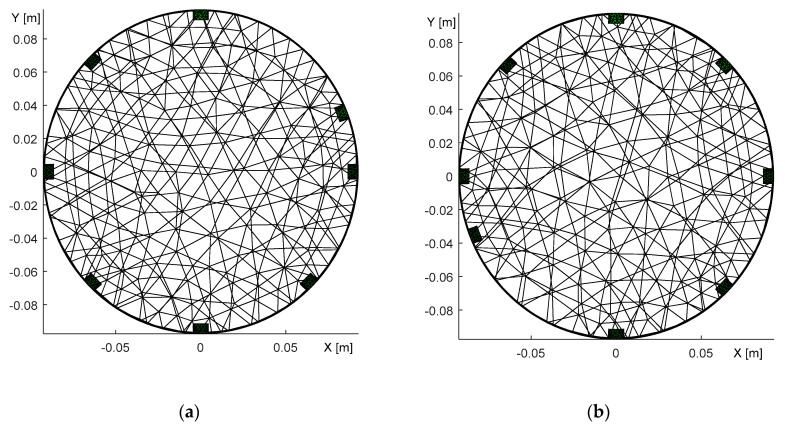
A top view of the optimized domain, with the electrode misplacement obtained through oppositesensing. The images display the shifted (**a**) 2nd electrode; (**b**) 6th electrode.

In addition to optimizing the electrode misplacement by means of adjacent driving, we performed the experiment also via the opposite stimulation pattern; the results are shown in Figure 14.

**Figure 15:**
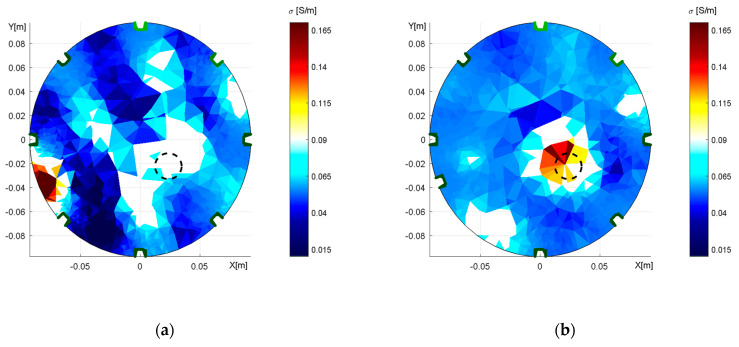
The conductivity distribution, shown to illustrate the misplacement effect relating to: (**a**) wrong equidistant placement of the 6th electrode, and (**b**) the correctly shifted 6th electrode.

Figure 14 characterizes the duality of the optimized model, obtained with the opposite sensingdataset. In the first image (Figure 14a), the 2nd electrode was placed in the angle of 68° after initially taking the angle of 45°; both angles are with respect to the 1st electrode. The second image then displays the 6th electrode at 248° (Figure 14b), the original position having been 225°. Both of the FEM models were verified via the forward task, and the simulated voltages exhibited almost identical values. Such a result allows us to conclude that opposite sensingwas not a suitable choice for investigating the electrode misplacement. Thus, the effect of inaccurate electrode placement was reconstructed only with adjacent driving, as is obvious from Figure 15.

Figure 15a displays an incorrect placement of the electrode and the resulting wrong localization of the inhomogeneity in the region of interest. By comparison, Figure 15b comprises the conductivity image reconstructed with the optimized model; the inhomogeneity is well localized. The randomly distorted area of conductivity in the reconstructed images may have arisen from insufficient resolution given by a limited number of active electrodes.

### 3.4. Error Evaluation

To evaluate the conductivity distribution accuracy, we sampled the reconstructed images at the resolution of 256 × 256 pixels. The total error was calculated via the relative root mean square error formulated through the following equation [50]:

(22) $$\;RRMSE(\sigma_{px})=\sqrt{\frac{\sum_{i=1}^{px}{\left(\sigma (i)-\sigma_{orig}(i)\right)}^{2}}{\sum_{i=1}^{px}\left(\sigma_{orig}(i\right){)}^{2}}}\cdot 100,$$

where *RRMSE(σ_px_)* is the total error in the reconstructed image, *px* denotes the number of pixels, *σ*(i) represents the reconstructed conductivity at a pixel of the sampled image, and *σ_orig_*(i) denotes the original conductivity given by the FEM model reflecting the real measurement setup.

The area of the inhomogeneous object was computed via comparison with the FEM model, which characterized the setup of the experiment and the reconstructed conductivity distribution. To evaluate the inhomogeneity space, we preset the threshold at 66% of the maximum conductivity in the image. This experimentally established threshold value facilitates effective suppression of the background conductivity and allows us to avoid losing the inhomogeneity-related information. The inhomogeneity area ratio was evaluated via the equation:

(23) $$\;IAR_{0.66}=\frac{\sum_{i=1}^{px}\sigma_{Inv}{\left(i\right)}_{}}{\sum_{i=1}^{px}\sigma_{Fwd}{\left(i\right)}_{}},$$

where *IAR*_0.66_ denotes the area ratio between the real and reconstructed conductivities in the heterogeneous object, *σ_Inv_*(i) represents the conductivity in the reconstructed image, and *σ_Fwd_*(i) stands for the original conductivity distribution expressed by the FEM model.

The discussed classifications enable us to evaluate the impact of the domain shape optimization process with respect to the reconstructed conductivity. The relevant values, as related to the inverse imaging, are summarized in Table 2.

The results of *RRMSE(σ_px_)* indicate an error decrease in the patterns, as follows: adjacent 6.16%, due to a more accurate position of the inhomogeneity; and opposite 11.58%, with the deformation-induced rotation of the inverse image suppressed. In adjacent driving, the inhomogeneity area ratio for the desired model was reduced by 0.637 in the complete image; the inhomogeneity area, however, exhibited a larger size, the difference being 0.11. In the opposite option, we reached a significant improvement in terms of both the complete image (by 2.443) and the inhomogeneity area (from 0.274 to 0.763). The results thus indicated that opposite driving is more sensitive to the shape deformations.

The effect of an incorrect electrode placement is outlined in Table 3, as follows:

Table 3 shows the overall error of the reconstructed image (Figure 15), which improved by 12.69% when the optimized model was employed. The inhomogeneity area ratio in the object space equaled 0.667 in the shifted 6th electrode but did not match the inhomogeneity in the incorrect equidistant setup. The *IAR* of the whole image increased by 1.641 in the relevant FEM model.

## 4. Hardware and Software

To carry out the experiment, we used the following devices and software:

- CPU: Intel Core i3-6098P (3.6 GHz); 16 GB RAM; operating system: Windows 10 (x64);
- Matlab R2016b (x64); EIDORS version 3.9;
- Keysight 34450A multimeter; Agilent DSO-X 3014A oscilloscope;
- PLI EIT system: version 0.1.

## 5. Discussion

The initial conductivity was optimized by using a water medium, with the relevant initial value established between 53.7 and 54.9 mS/m in the circular and the elliptical domains (Table 1). The computational process to execute the task took 7 s at the maximum.

The Nelder–Mead algorithm also facilitated resolving the shape deformation, where the deformation of the circular domain was determined in the axes X and Y. For this reason, we tightened the laboratory model in a clamp to obtain a deformation of 2%. Subsequently, we modified the domain and estimated the diameters of the axes via optimization, yielding X = 18.62 cm and Y = 19.35 cm in the adjacent driving and X = 18.66 cm and Y = 19.35 cm in the opposite pattern. Assuming the real axis dimensions of X = 18.6 cm and Y = 19.4 cm, these results are acceptable. The optimization procedure took 205–250 s. The effect of inaccurate domain boundaries was demonstrated by conductivity imaging based on elliptical domain measurement, performed properly for the accurate model and incorrectly for the circular one (Figure 9 and Figure 10). The imprecise shape of the domain led to wrong localization of the inhomogeneity with respect to the original position of the object. The optimized model reduced the overall conductivity distribution errors of 6.16% and 11.58% in the adjacent and opposite patterns, respectively. The inhomogeneity area ratio relating to the localization defined by Equation (23) rose from 0.658 to 0.768 (11.0%) in the adjacent driving and significantly improved in the opposite pattern, increasing from 0.274 to 0.763 (48.9%). The overall conductivity-related inhomogeneity area ratio in the correct model decreased from 2.542 to 1.905 in the adjacent and from 5.947 to 3.504 in the opposite patterns. The results indicate that the opposite pattern was significantly more sensitive to shape deformations (Table 2).

The optimization also involved evaluating the electrode misplacement, this being the source of inaccurate conductivity imaging. In the given context, we prepared the model with the shifted position of the 6th electrode and then measured the dataset via adjacent and opposite driving. The optimization was performed in homogeneous conditions, with the convenient results obtained through the adjacent pattern and the non‑acceptable duality produced by the opposite option. The duality generated two different outcomes, namely, a shift of the 2nd and the 6th electrodes, which are interchangeable in the forward task (Figure 14). Based on this scenario, we concluded that opposite driving is not suitable for optimizing the electrode misplacement. The electrode misplacement effect was verified via reconstruction. The conductivity images show that the inhomogeneity was correctly localized with the optimized model. The conductivity error over the image dropped by 12.69%, and the inhomogeneity localization was improved markedly, from zero match to a conformity of 0.667 (Table 3). The computational time for the electrode misplacement oscillated between 90 and 110 s.

## 6. Conclusions

This article examined and discussed several factors that affect conductivity imaging, including but not limited to initial conductivity, shape domain deformation, and electrode misplacement. In the introductory chapter, a brief overview of the prior research is outlined. Based on the previous work and the problems we encountered when correlating the laboratory tomograph with the numerical model, we prepared optimization via the Nelder–Mead algorithm. The real dataset was created by means of measurements exploiting single-source driving, or, more concretely, the adjacent and the opposite patterns on the 8-electrode tomograph. For this reason, we employed a precise, low impedance EIT system. The optimization algorithm processed the forward and the inverse tasks by utilizing the EIDORS library. The three-dimensional parametric FEM models with approximately 15,000 elements were generated via Netgen (Figure 5). The results of the optimization were evaluated by the conductivity error rate (Equation (22)) and the inhomogeneity area ratio (Equation (23)) in Table 2 and Table 3.

As regards the actual impact on the consumers, optimizing the properties of the mathematical model significantly improves the image reconstruction accuracy. In this context, we propose a novel approach to integrating the properties of the mathematical model with those of the physical one. This paper also outlines a viable procedure for optimizing a model of an experimental impedance tomograph by utilizing a simple measurement based on Matlab Optimization Toolbox and the EIDORS platform. By extension, Figure 10 shows that opposite feeding and sensing render the conductivity distribution more sensitive to undesired variations of the domain shape, and it also demonstrates that the method is not usable in the optimization of electrode positions.

At present, the optimizing process solves only one parameter during a run. Another limitation rests in an increasing number of degrees of freedom (by including more parameters to optimize simultaneously), an effect that markedly affects the computational intensity. Although the paper does not discuss experiments involving the simultaneous optimization of two or more parameters of the mathematical model, currently available results indicate that such an option features insufficient stability and may not yield the desired outcome. Yet another problem is inadequate measurement accuracy, where the optimization error can be caused by not only the voltage and phase shift uncertainties but also high noise levels. The future efforts will be directed towards the following aspects or procedures: improving the accuracy of the results achievable with the tomograph, using better parameterizable models and a different mesh generator; reducing the computational intensity and cost; and including the contact impedance, which is important in the measurement of current-carrying electrodes. We also intend to focus on producing a novel and more precise version of the low impedance EIT data acquisition unit, designed to feature a programmable and easily extendable switch with low on-state impedance.

## Figures and Tables

**Table 1:** The results: the optimization of the initial conductivity.

Current Pattern	Domain Shape	Conductivity [mS/m]
Adjacent	Circular	54.4
Adjacent	Elliptic	53.7
Opposite	Circular	54.9
Opposite	Elliptic	54.2

*I* = 2.002 mA; *f* = 1007 Hz.

**Table 2:** The image errors in the individual domain shape deformations.

Current Pattern	Domain Shape	*RRMSE* (*σ_px_*) [%]	*IAR*_0.66_ [-]
Adjacent	Circular	34.59	0.658/2.542
Adjacent	Elliptic	28.43	0.768/1.905
Opposite	Circular	51.99	0.274/5.947
Opposite	Elliptic	40.41	0.763/3.504

The measurement was performed on an elliptically deformed domain.

**Table 3:** The electrode placement error in the conductivity reconstruction.

Electrode Position	*RRMSE*(*σ_px_*) [%]	*IAR*_0.66_ [-]
Equidistant	52.73	0.000/1.483
6th shifted	40.04	0.667/3.124

The measurement was performed with the adjacent stimulation pattern on an elliptically deformed domain, utilizing the non-equidistant electrode setup (shifted 6th electrode).

## Data Availability

Not applicable.
